# Laser Doppler Assessment of Vasomotor Axon Reflex Responsiveness to Evaluate Neurovascular Function

**DOI:** 10.3389/fneur.2017.00370

**Published:** 2017-08-14

**Authors:** Marie Luise Kubasch, Anne Sophie Kubasch, Juliana Torres Pacheco, Sylvia J. Buchmann, Ben Min-Woo Illigens, Kristian Barlinn, Timo Siepmann

**Affiliations:** ^1^Department of Neurology, Carl Gustav Carus University Hospital, Technische Universität Dresden, Dresden, Germany; ^2^Center for Rare Diseases, Children’s Hospital, Carl Gustav Carus University Hospital, Technische Universität Dresden, Dresden, Germany; ^3^Division of Health Care Sciences, Dresden International University, Dresden, Germany; ^4^Department of Neurology, Charite University Medicine, Berlin, Germany; ^5^Department of Neurology, Beth Israel Deaconess Medical Center, Harvard Medical School, Boston, MA, United States

**Keywords:** laser Doppler, imaging, flowmetry, skin, neuropathy, assessment, axon

## Abstract

The vasomotor axon reflex can be evoked in peripheral epidermal nociceptive C-fibers to induce local vasodilation. This neurogenic flare response is a measure of C-fiber functional integrity and therefore shows impairment in patients with small fiber neuropathy. Laser Doppler flowmetry (LDF) and laser Doppler imaging (LDI) are both techniques to analyze vasomotor small fiber function by quantifying the integrity of the vasomotor-mediated axon reflex. While LDF assesses the flare response following acetylcholine iontophoresis with temporal resolution at a single defined skin point, LDI records flare responses with spatial and temporal resolution, generating a two-dimensional map of superficial blood flow. LDF is characterized by a high intra- and interindividual measurement variability, which is smaller in LDI due to its spatial resolution. Nevertheless, LDI still lacks standardized methods for image analysis. Consequently, use of the technique currently remains on an experimental level. Here, we sought to review the current literature on laser Doppler assessment of vasomotor function and discuss potential future applications of established techniques as well as those that are still experimental.

## Introduction

In small fiber neuropathy, small somatic fibers and/or autonomic fibers are affected. Small fibers are unmyelinated C-fibers or thinly myelinated A-delta fibers that mediate thermal sensation, pain, and autonomic function, such as blood vessel and sweat gland control. A selective damage to these peripheral nerve fibers can be caused by a number of conditions and therefore comprises a variety of pathophysiologically heterogenous disorders, among them diabetes, neurodegenerative diseases, or paraneoplastic syndromes. Small fiber neuropathy is associated with increased cardiovascular morbidity and mortality, therefore early diagnosis and continued assessment of disease progression is important (1, 2). Assessment of cutaneous vasomotor function is a promising way to diagnose and monitor small fiber neuropathy. However, only few techniques are available.

Based on the assessment of the Doppler shift of backscattered laser light by moving red blood cells (3), change in cutaneous blood flow due to vasoconstrictive or vasodilating stimuli can be quantified using single-point laser Doppler Flowmetry (LDF) or the two-dimensional laser Doppler imaging (LDI). To test the neurogenic control of cutaneous blood vessels, these techniques are combined with iontophoresis of a cholinergic agent, such as acetylcholine, to allow assessment of the integrity of the neurogenic axon reflex as a way to investigate small nerve fiber function (4, 5).

Laser Doppler flowmetry investigates vasomotor axon reflex response with temporal resolution in fixed points of the skin. It is widely used in research but its clinical use is limited due to a high intra- and interindividual variability. LDI assesses the vasodilatory response after acetylcholine iontophoresis using spatial and temporal resolution, thereby reducing variability. However, it has not attained widespread use, and only few clinical studies have used this technique to assess neurovascular dysfunction (Table 1).

**Table 1:** Comparative table on the two techniques.

	Laser Doppler flowmetry	Laser Doppler imaging
Flare response	– Temporal resolution at a single defined skin point with a predefined distance from the delivery capsule of acetylcholine – Illumination of tissue sample with single-frequency laser light – Perfusion estimation by processing the frequency distribution of the backscattered light	– Spatial and temporal resolution, generating a two-dimensional map of superficial cutaneous perfusion over time – Imaging instrument scans skin area with a laser beam to record images, as a map of single-point cell data – Images reflect perfusion values of each measured cell
Characteristics	– Not applicable for clinical diagnostic of neuropathy induced vasomotor dysfunction because of high intra- and interindividual measurement variability – Wide use in research to differentiate between patients with peripheral neuropathy and healthy individuals	– Smaller variability due to its spatial resolution – Applicable as clinical diagnostic tool to assess vasomotor dysfunction due to small fiber neuropathy – Clinical use is limited due to lack of standardized image analysis methods – Currently remains on an experimental level of clinical diagnosis

This review provides a summary of the current literature on laser Doppler assessment of vasomotor function and critically discusses the utility and drawbacks of available and experimental techniques in research and clinical practice as well as potential future implications.

## Assessment of Vasomotor Axon Reflex Response

### The Vasomotor Axon Reflex

**Figure 1:**
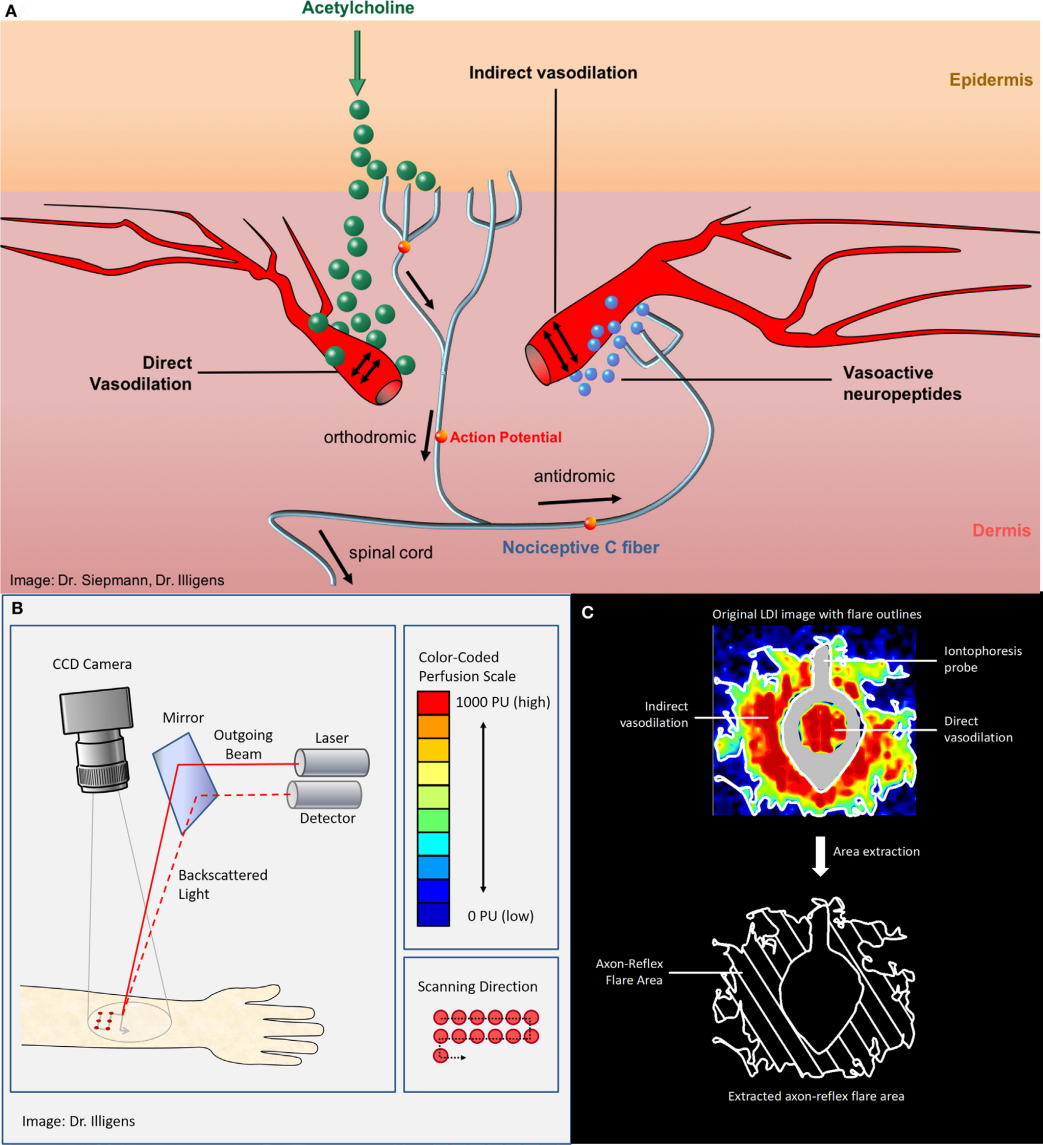
**(A)** The vasomotor axon reflex: iontophoresis of acetylcholine results in stimulation of peripheral epidermal nociceptive C-fibers and thereafter indirectly results in local vasodilatation. The topical stimulation depolarizes unmyelinated C-fibers in the skin and results in afferent action potentials that are conducted orthodromically toward the spinal cord. At branching points of the nerve, the impulse continues antidromically, ending at arterial dermal vessels adjacent to the initial stimulation point. There, the axon reflex induces the release of vasoactive substances, such as substance P and calcitonin gene-related peptide, from nerve terminals and leads to vasodilation. **(B)** Laser Doppler imager setup and principle of measurement. **(C)** The upper panel shows the color-coded laser Doppler imaging perfusion data with iontophoresis probe framing direct vasodilation induced by direct stimulation as well as axon reflex-mediated enhancement of blood flow (“flare”) in an adjacent skin area surrounding the probe (“indirect flare”). The lower panel shows the extracted axon reflex flare, which provides a measure of functional integrity of those nociceptive C-fibers that mediate the vasomotor axon reflex upon activation through acetylcholine.

Specific examination of quality and integrity of peripheral autonomic nerve fibers can be performed with vasomotor (neurovascular endothelial function) and sudomotor (sweat gland function) tests that are usually only performed in specialized academic centers. In the beginning of the twentieth century, the term “axon reflex” has been described in vasomotor fibers that were studied and investigated afterward in different clinical settings (6, 7). The axon reflex can be evoked directly by pharmacological (iontophoresis of 10% acetylcholine), thermal, electrical, or mechanical stimuli. The topical stimulation depolarizes unmyelinated C-fibers in the skin and results in afferent action potentials that are conducted orthodromically toward the spinal cord. At branching points of the nerve, the action potential continues antidromically, ending at dermal blood vessels adjacent to the initial stimulation point. This triggers the release of vasoactive substances, such as substance P and calcitonin gene-related peptide, from nerve fiber terminals and leads to a vasodilatory response (7–9) (Figure 1). The extended area of cutaneous vasodilation following acetylcholine iontophoresis is mediated indirectly through the axon reflex, indicating the functional integrity of vasomotor C-fibers (7, 8).

### Iontophoresis of Acetylcholine to Induce Axon Reflex-Mediated Blood Flow

Iontophoresis of vasoactive substances *via* a drug-delivering capsule is a common technique for studying pharmacological aspects of the microvascular function (10). Particularly iontophoresis of acetylcholine is used to evoke axon reflex–mediated increase in blood flow, also referred to as flare response (7, 11). Iontophoresis of acetylcholine stimulates endothelium-dependent production of substance P, calcitonin gene-related peptide, and nitric oxide leading to microvascular vasodilation. Therefore, the detected flux beneath the iontophoresis capsule is referred to as direct response (12, 13). In contrast, the detected flux next to the iontophoresis capsule, called indirect response, is axon reflex mediated and therefore represents a measure of small nerve fiber function (7, 11, 14).

During the past 10 years, iontophoresis technique has been used in various experimental settings but to date, there are no consistent iontophoresis protocols throughout the vasomotor studies (15, 16). Current density, dose, and acetylcholine concentration differ between studies, making the comparison of study results difficult (17–19).

In addition, the axon reflex can be evoked by a wide range of physiologic stimuli such as heat, cold, mechanical distension, and UV light (20, 21). However, in clinical research, iontophoresis of acetylcholine has been widely established due to its precision in delivering a predefined dosage of the compound. Moreover, the technique may be superior to others due to its capacity to mediate a neurogenic blood flow response which has been shown to be mediated neither by phenomena secondary to iontophoresis nor endothelium-mediated vasodilation (22–25).

## Laser Doppler Assessment

Axon reflex-based tests of peripheral small nerve fiber function assess neurogenic response of cutaneous blood vessels to vasodilatory stimuli and can be quantified by LDF and LDI (14).

### Single-Point LDF

Laser Doppler flowmetry uses a single-frequency (monochromatic), coherent laser light source on a fixed area of the skin. Processing the frequency distribution of the backscattered light allows an estimation of the microvascular blood perfusion (3). LDF quantifies the vasodilator response following acetylcholine iontophoresis using temporal resolution at a single and defined skin point, with a predefined distance from the delivery capsule of acetylcholine (4). The test thereby allows detecting cutaneous perfusion differences between patients with peripheral neuropathy and healthy individuals (1).

### Two-dimensional LDI

Laser Doppler imaging constitutes a newer axon reflex-based measurement method, which records the vasomotor axon reflex-mediated flare response with spatial and temporal resolution and generates a two-dimensional map of the cutaneous blood flow (26). The imaging instrument scans the desired, predefined skin area with a laser beam at a fixed distance from the surface of the skin, allowing to generate a perfusion map of a heterogeneous tissue (2, 27). In this technique, the principal task of the laser Doppler imager is to record images, as a map of single-point cell data (2). These images reflect relative values of each measured cell, measured in perfusion units. When a cell’s perfusion unit exceeds a threshold, fixed at an arbitrary level, a flare response is registered. These baseline characteristics do not change across analysis techniques, but the definition of the appropriate threshold have been different between each analysis method (2). The presence of different analysis techniques to measure the extent of LDI axon reflex-induced flare area limits its consistent application and generalization of results. The search for the best technique is, therefore, objective of ongoing clinical studies.

#### LDI Axon Reflex Flare Area Test

Laser Doppler imaging measures the microvascular blood flow sequentially over a defined skin area, representing skin blood flow two-dimensionally over time (28). Thus, LDI allows creation of a topographical map of skin perfusion by estimating the extent of the axon reflex-based flare area following the vasodilator response after acetylcholine iontophoresis (9). Using standardized conditions, the laser Doppler perfusion imager obtains over a predefined area of interest baseline images to quantify the resting blood flow, followed by images taken during and after iontophoresis. Obtained perfusion images are analyzed using a color perfusion code, where dark blue represents the lowest perfusion value, and red the highest perfusion value displayed (4). Compared to the single-point blood flow measurement of LDF, LDI allows visualization of the size, intensity, and shape of the axon reflex-induced hyperemic flare area and calculation of the average perfusion (14, 29). The subtraction of the direct dilation area gives a measure of the LDI detected flare area (Figure 1). Since the flare depends on an intact axon reflex response in peripheral C-fibers, patients with neuropathy show a decreased flare area as measured by LDI (14). Typically, the axon reflex-mediated local blood flow presents an irregular shape, rather than a homogeneous circle. This is a challenge when using LDF, since probes are placed at a fixed distance from the iontophoresis capsule, and therefore can be easily placed over a less innervated skin area resulting in a large coefficient of variation (7, 11, 14, 30). Thus, LDI might be more suitable for clinical evaluation of small fiber neuropathies (14, 31, 32).

##### Available Blood Flow Analysis Techniques

There are several techniques available to analyze the axon reflex-induced vasodilatory response following acetylcholine iontophoresis. All analyzing techniques have in common that a baseline perfusion threshold is predetermined initially and used as reference for subsequent perfusion measurements.

###### Flare Area Method

The flare area method sets the threshold by averaging the maximum perfusion reaction of all cells. Thereafter, the axon reflex flare is determined by the number of cells above the set threshold after acetylcholine iontophoresis subtracted from the number of cells above the threshold before the iontophoresis (2):

$$[\text{axon}-\text{reflex flare}=({\text{Flare Area}}_{\text{iontophoresis}}-{\text{Flare Area}}_{\text{baseline}})].$$

###### Baseline Perfusion Method

The baseline perfusion method defines the blood flow threshold as two standard deviations of the baseline perfusion. Thereafter, the total axon reflex-mediated flare area is calculated based on the total number of cells exceeding the defined threshold (2).

###### Resting Blood Flow Method

After acetylcholine iontophoresis the axon reflex-mediated flare response is represented by the measurement of the total number of cells exceeding the previous identified blood flow threshold (2, 14). Compared to resting blood flow, axon reflex-mediated flare response was defined as an increase of three times of the baseline blood flow value (14).

## Limitations and Drawbacks

Both LDF and LDI should be applied under constant temperature, humidity, and light conditions to ensure reliable measurements. The distance to the tested skin area should be constant as well in all measurements as it influences the outcome of the imaging processes of both techniques. A calibration of each device before starting the measurement is recommended. Therefore, the use of both methods in bedside testing is challenging from the clinical point of view. Moreover, the LDI as well as the LDF technique require a thorough preparation of the studied skin area. Hairs should be removed from the skin, and skin scales should be removed carefully to avoid any disturbances in the imaging processes. The use of both methods on irritated skin is not recommended. In addition, for LDI, movement of the scanned area has to be avoided since this will introduce movement artifacts in the recording, limiting quality of the data obtained.

Laser Doppler flowmetry assessment is characterized by a high intra- and interindividual variability and is limited by technically demanding settings. Since most investigators agree that it lacks sensitivity to detect vasomotor neuropathy in individuals during clinical routine diagnosis, LDF is being limited to specialized clinical centers with specific demands and research studies (26, 29, 33–35). Furthermore, LDF cannot determine the extent of the flare area, since the blood flow change is measured in single points at a predetermined distance from the site of iontophoresis (4, 23, 33, 34).

Using the LDI technique in clinical or research setting requires training. It is recommended to set up the LDI equipment before acetylcholine iontophoresis. The LDI needs to be placed at an appropriate distance over the iontophoresis capsule parallel to the skin before starting the imaging process. The presence of different analysis techniques to measure the extent of LDI axon reflex-induced flare area limits its consistent application and generalization of results. Most importantly, to improve utility standardization of image analyses presets is necessary. Therefore, studies to define parameters relevant to diagnostic discrimination such as sensitivity and specificity are needed. Especially, a set of normative data gained in studies in large populations of healthy subjects compared with patients with neuropathy should form the basis for future application. Furthermore, technical independence from environmental factors such as light, temperature, and humidity would improve its application. Until now, application of LDI axon reflex flare area test remains on an experimental level in clinical diagnostics and is limited to specialized centers (2, 14, 26, 31, 32).

## Laser Doppler-Based Assessment of Neurovascular Function in Clinical Research

Even though clinical studies assessing these tests are still rare, some studies have been published proving their utility. It has been suggested that LDI is a simple method to detect early neuropathy in subjects with type 2 diabetes and may have some value as an endpoint to assess therapeutic interventions aiming to prevent or reverse C-fiber dysfunction (4). Furthermore, laser Doppler devices used to measure C-fiber axon reflex showed impairment in both patients with type 1 and type 2 diabetes (8). It is possible that functional abnormalities in diabetic peripheral neuropathy are not homogeneous, however whether C-fiber subclasses are differentially involved in type 1 and type 2 diabetes mellitus remain speculative and warrant further studies (5).

Laser Doppler imaging has shown a high sensitivity for diabetic mixed fiber neuropathy detection and could be performed with immediate results (36). Its reproducibility was also confirmed in healthy subjects (37). LDF had been studied in familial dysautonomia and has helped to understand the relationship between receptors and innervation in disorders of autonomic dysfunction (38). It has also been used to detect the onset of small fiber neuropathy also in transthyretin-related familial amyloid polyneuropathy (39).

In neurodegenerative disorders, reduced laser Doppler blood flow in patients with Huntington’s disease supports the hypothesis that autonomic nervous system dysfunction is part of the pathogenesis of Huntington’s disease (40). In patients with Alzheimer’s disease, LDI has shown a significantly attenuated skin vascular response following iontophoresis of acetylcholine, indicating abnormalities in receptor/signal transduction, supporting the idea that Alzheimer’s disease might be a systemic disorder (41). Recently, reduced vasoconstrictive response of cutaneous blood vessels of patients with Parkinson’s disease compared to healthy subjects has been shown using LDF, indicating a significant impairment of vasomotor function in these patients as a manifestation of cutaneous autonomic neuropathy (42, 43). Importantly, severity of impaired vasoconstrictive response increased in advanced disease stages (44).

## Conclusion and Perspective

Assessment of microvascular function in peripheral small fiber neuropathy is useful to diagnose vasomotor dysfunction and supplements other well-established autonomic function tests, such as tilt-table testing and the measurement of heart rate variability. Hence, vasomotor axon reflex-mediated flare measurement techniques have been gaining importance in recent years not only in research studies but also in specialized academic and clinical centers.

Laser Doppler imaging presents increased accuracy and reproducibility when compared to the single-point LDF technique by using temporal and spatial resolution. However, standardized blood flow image analysis warrants further validation in larger study groups before the technique can be established in clinical practice.

## Author Contributions

MK: conception and drafting the manuscript. AK, JP, SB, and KB: reviewing the manuscript for intellectual content. BI: creating figures and reviewing the manuscript for intellectual content. TS: conception, creating figures, and reviewing the manuscript for intellectual content.

## Conflict of Interest Statement

The authors declare that the research was conducted in the absence of any commercial or financial relationships that could be construed as a potential conflict of interest.
